# Functional Connectivity Evoked by Orofacial Tactile Perception of Velocity

**DOI:** 10.3389/fnins.2020.00182

**Published:** 2020-03-06

**Authors:** Yingying Wang, Fatima Sibaii, Rebecca Custead, Hyuntaek Oh, Steven M. Barlow

**Affiliations:** ^1^Neuroimaging for Language, Literacy and Learning Laboratory, Department of Special Education and Communication Disorders, University of Nebraska-Lincoln, Lincoln, NE, United States; ^2^Center for Brain, Biology and Behavior, University of Nebraska-Lincoln, Lincoln, NE, United States; ^3^Nebraska Center for Research on Children, Youth, Families and schools, University of Nebraska-Lincoln, Lincoln, NE, United States; ^4^Biomedical Engineering, University of Nebraska-Lincoln, Lincoln, NE, United States; ^5^Communication Neuroscience Laboratory, Department of Special Education and Communication Disorders, University of Nebraska-Lincoln, Lincoln, NE, United States

**Keywords:** functional connectivity, orofacial, pneumotactile stimulation, fMRI, saltatory velocity, cortical representation

## Abstract

The cortical representations of orofacial pneumotactile stimulation involve complex neuronal networks, which are still unknown. This study aims to identify the characteristics of functional connectivity (FC) evoked by three different saltatory velocities over the perioral and buccal surface of the lower face using functional magnetic resonance imaging in twenty neurotypical adults. Our results showed a velocity of 25 cm/s evoked stronger connection strength between the right dorsolateral prefrontal cortex and the right thalamus than a velocity of 5 cm/s. The decreased FC between the right secondary somatosensory cortex and right posterior parietal cortex for 5-cm/s velocity versus all three velocities delivered simultaneously (“All ON”) and the increased FC between the right thalamus and bilateral secondary somatosensory cortex for 65 cm/s vs “All ON” indicated that the right secondary somatosensory cortex might play a role in the orofacial tactile perception of velocity. Our results have also shown different patterns of FC for each seed (bilateral primary and secondary somatosensory cortex) at various velocity contrasts (5 vs 25 cm/s, 5 vs 65 cm/s, and 25 vs 65 cm/s). The similarities and differences of FC among three velocities shed light on the neuronal networks encoding the orofacial tactile perception of velocity.

## Introduction

The human somatosensory system decodes tactile stimuli from peripheral sensory receptors through a complex process involving interactions between bottom-up thalamocortical and top-down corticocortical/cortico-thalamo-cortical pathways (Avivi-Arber et al., 2011; Lundblad et al., 2011; Zembrzycki et al., 2013; Hwang et al., 2017). Studies of cortical representations of tactile stimulation of different body parts have identified the primary (SI) and secondary (SII) somatosensory cortices, as well as the supplementary motor area responsible for sensory processing (Ibáñez et al., 1995; Backes et al., 2000; Grodd et al., 2001; Backlund et al., 2003; Paus et al., 2006; Backlund Wasling et al., 2008; Bjornsdotter et al., 2009; Ackerley et al., 2012; Zembrzycki et al., 2013; Akselrod et al., 2017; Custead et al., 2017; Oh et al., 2017; Yeon et al., 2017). SI, which is located in the postcentral gyrus, processes complex information about the location, velocity, and other characteristics of tactile stimulation from the thalamus through the thalamocortical axons. Damage to the SI (e.g., by stroke, traumatic brain injury, etc.) could result in orofacial sensory and motor deficits; the recovery of such damage to the sensorimotor system requires characterizing the neuronal connections (both structural and functional connectivity) (Nudo, 2013). Therefore, the neuronal connections among cortical regions are critical for understanding the cortical plasticity after injuries to the somatosensory system.

Tactile sensation is detected by cutaneous mechanoreceptors in the skin and is then projected to afferent neurons or sensory nerves, via the spinal cord, toward the central nervous system (Jenkins and Lumpkin, 2017). Pacinian corpuscles, a type of cutaneous mechanoreceptors that usually detect rapid vibrations (about 200–300 Hz) in both glabrous and hairy skin (e.g., palm and arm, respectively), were considered to be virtually absent from the facial skin based on psychophysical methods (Barlow, 1987), and the cutaneous mechanoreceptors in the facial skin have high densities and are slow adapting, with small receptive fields (Johansson and Olsson, 1976; Johansson et al., 1988).

Vibrotactile adaption has been observed in both the hands and face (Hollins et al., 1991). Individual mechanoreceptors in the facial skin responded differently to brush stimuli moving at different velocities (Edin et al., 1995). Nevertheless, it has been argued that the central nervous system might not be able to decode velocity of movement across the skin in humans (Edin et al., 1995). However, animal studies have suggested that rat SI neurons could process complex tactile stimuli such as direction and velocity of motion (Moore et al., 1999; Krupa et al., 2001; Ferezou et al., 2007; Tomita et al., 2012; Zembrzycki et al., 2013). Furthermore, neuroimaging studies also indicated that there are different cortical representations for different tactile stimuli in humans (e.g., location, type of motion, direction, velocity, etc.) (Reed et al., 2004; Miyamoto et al., 2006; Backlund Wasling et al., 2008; Eickhoff et al., 2008; Bjornsdotter et al., 2009; Moulton et al., 2009; Avivi-Arber et al., 2011; Grabski et al., 2012; Huang et al., 2012; Khoshnejad et al., 2014; Yang et al., 2014; Custead et al., 2015, 2017; Hwang et al., 2017; Oh et al., 2017; Yeon et al., 2017).

The cross-modality plasticity theory suggested that somatosensory stimuli could evoke neural responses to promote learning of new motor skills (Sanes and Donoghue, 2000; Nasir et al., 2013; Ladda et al., 2014) and performing motor tasks more accurately (Pearson, 2000). The integration of the orofacial sensory and motor system has been suggested to be critical for motor learning and motor control for sucking, swallowing, and producing speech sounds (Barlow and Bradford, 1996; Barlow, 1998; Sessle et al., 2005, 2007; Barlow and Estep, 2006; Barlow and Stumm, 2010; Smith, 2016). If passive pneumotactile stimulation could effectively evoke changes in the neuronal connections and positively impact motor learning, there may be a paradigm shift in early neurorehabilitation protocols to improve functional recovery after brain injury (e.g., due to stroke, traumatic brain injury, etc.).

The cortical representations of moving tactile stimulation have mostly been investigated on the hand. Brushing over the right thumb once every one and a half second and using electric stimuli to the median nerve in seven healthy participants, Lin et al. identified the different shapes of waveforms of somatosensory evoked fields in SI using magnetoencephalography (MEG) (Lin and Kajola, 2003). A functional magnetic resonance imaging (fMRI) study compared active touch, self-touch, and passive touch of both the glabrous palm and hairy arm, using a stroking velocity between 6 and 8 cm/s and demonstrated specialization of cortical regions for processing of somatosensory information (Ackerley et al., 2012). They found that moving tactile stimulation of the glabrous palm activated more extensive cortical areas of the right SI (subarea Brodmann area BA 3, contralateral) than that of the hairy arm. Moreover, active stroking evoked positive blood-oxygenation level dependent (BOLD) signals in the left SI (ipsilateral), whereas passive stroking evoked negative BOLD signals. More recently, a fMRI study identified the left SI, left superior temporal gyrus (STG), and the left precentral gyrus (preG) as being involved in encoding saltatory pneumotactile velocity stimulation of the glabrous hand, using stimuli at 5, 25, and 65 cm/s (Oh et al., 2017). The velocity of 25 cm/s evoked the most extensive BOLD signal among all velocity settings.

However, for the face, the cortical representations of moving tactile stimulation have not been well studied. Not knowing the neural subtracts of perceiving moving stimulation on the face has limited our understanding of velocity and directional encoding in the sensory domain. In our previous fMRI study, we identified a putative neural somatosensory velocity network with bilateral SI, bilateral cerebellum, bilateral middle occipital gyrus, left MI, right SII, right STG, and right SMG, right inferior frontal gyrus (IFG), which had not been reported previously (Custead et al., 2017). Custead et al. used a univariate generalized linear model (GLM) to determine brain regions with specific responses to the pneumotactile stimulation at each voxel. The univariate GLM approach assumes that each voxel in the brain is functionally specialized rather than functionally integrated (Stephan et al., 2006). However, this perspective limits our understanding of how different brain regions communicate with each other, which is essential for understanding complex neuronal networks (Tononi et al., 1998). Task-based functional connectivity (FC) measured by evaluating the correlation between time series of BOLD signals among brain regions does not measure structural connections (e.g., axonal projections), but represents the functional coupling between two or more spatially or anatomically distinct areas of the brain (Stevens, 2009; Hermundstad et al., 2013). To date, little is known about the functional connectivity (FC) evoked by the orofacial tactile perception of velocity.

The present study therefore aimed to identify the characteristics of FC evoked by pneumotactile stimuli, at three saltatory velocities (5, 25, and 65 cm/s), on the non-glabrous lower face, as an extension to our previous work, in order to enhance our understanding of the neural networks responsible for encoding the velocity of tactile stimulation (Custead et al., 2017). We hypothesized that there would be different patterns of FC corresponding to the three velocities.

The pneumotactile stimulator used in this study (Galileo^TM^) overcomes the technical challenges of automatically applying tactile stimulation to the face without eliciting pain sensation during fMRI (Custead et al., 2017). A single chambered tactile cell (TAC-Cell) or multiple TAC-Cells of the Galileo system can be placed on both glabrous and hairy skin through adhesive tape collars, and are compatible with many neuroimaging techniques, such as MRI, functional near-infrared spectroscopy (fNIRS), MEG, and electroencephalography (EEG). The in-house computer software allows the Galileo system to deliver saltatory tactile stimuli at a variety of settings (amplitude, velocity, etc.). Unlike other pneumotactile stimulators (Dresel et al., 2008; Huang et al., 2012), the Galileo with TAC-Cells is easy to set up and program for various applications. This pneumotactile stimulator has been used to examine the neural subtracts of the human somatosensory system and has effectively activated SI, SII, and the PPC (Popescu et al., 2013; Custead et al., 2017; Oh et al., 2017).

Based on our previous work and other studies in the literature (Blatow et al., 2007; Huang et al., 2012; Custead et al., 2017), we here chose 10 regions-of-interest (ROIs), including the bilateral SI, SII, PPC, dorsolateral prefrontal cortex, and thalamus for hypothesis-driven ROI-to-ROI FC analysis, to examine whether the FC of our hypothesized networks differ across the three stimulation velocities. Then, four ROIs, including the bilateral SI and SII, were chosen for data-driven Seed-to-Voxel FC analysis to examine which cortical areas are functionally connected to either SI or SII, and whether this differ across the three stimulation velocities.

## Materials and Methods

### Participants

Twenty healthy, right-handed, native English-speaking adults (15 females), 18–30 years of age (mean ± SD: 22.3 ± 1.7), agreed to participate in the study after providing written informed consent. All participants reported the right hand as the preferred hand and had no history of neurological or psychiatric disorders, or any chronic illness or scheduled medications. The study was approved by the Institutional Review Board at the University of Nebraska-Lincoln.

### Stimulus Device

**FIGURE 1:**
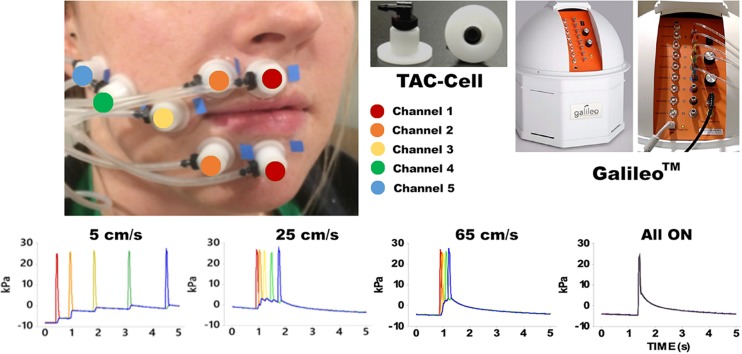
Shows the experimental configuration for the Galileo somatosensory stimulator with pneumatic velocity arrays aligned on the participant from the right philtral column to the right buccal face. White flanged surface of the TAC-Cell was adhered to skin surface with double adhesive colars and 7 TAC-Cells form 5 channels in five colors (red: channel 1 placed two TAC-Cells on the median upper and lower lips; orange: channel 2 placed two TAC-Cells next to the TAC-Cells of channel 1; yellow: channel 3 placed a TAC-Cells next to the channel 2; green: channel 4 placed a TAC-Cell next to channel 3; blue: channel 5 placed a TAC-Cell next to channel 4). The bottom four graphs show the time courses for each velocity and All ON.

Pneumotactile stimuli were delivered to the facial skin by a multichannel pneumatic amplifier and tactile array known as the Galileo Somatosensory^TM^ system (Epic Medical Concepts & Innovations, Inc., Mission, Kansas, KS, United States). The Galileo system utilizes TAC-Cells made from acetyl thermoplastic homopolymer, which uses tiny volumes of compressed air to deform the surface of the skin rapidly. The TAC-Cells are MRI-safe and incorporate a small capsule with a sealing flange. In Figure 1, the placement of seven TAC-Cells on a representative participant was shown from the upper and lower lips to the right lateral cheek of the face. Before using double-adhesive tape collars to secure each TAC-Cell, ten percent concentration of tincture of Benzoin was applied to the skin for improvement of adhesion. For each participant, the distances between each TAC-Cell were measured and taken into consideration for designing the velocity trains traversing in a repeating medial-to-lateral direction on the face. For all conditions, the Galileo system was programed to generate biphasic pulses with a duration of 60 ms, frequency of 1 Hz, 10 ms rise/fall, amplitude from −5 to 28 kPa (Custead et al., 2017; Oh et al., 2017). Our in-house software program generated air pressure pulses to five channels sequentially for 5, 25, 65 cm/s conditions and simultaneously for “All ON” condition. The Galileo system was located outside the MRI suite and delivered pneumotactile stimuli through polyurethane tubing into the MRI suite. Participants described the sensory experience as a moving sequence of discrete ‘taps’ or ‘raindrops’ on their lower face without any discomfort (Custead et al., 2017).

### Paradigms

**FIGURE 2:**
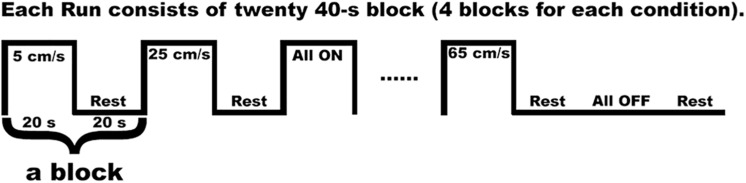
One run of the fMRI in-scan paradigm indicates a series of pneumotactile saltatory stimuli traversed the skin in a repeating medial-to-lateral direction. There are twenty 40 s long blocks in one run. Each block consisted of one 20 s block of task stimuli presentation and 20 s block of rest. There are five possible task blocks (5, 25, 65 cm/s, All ON, All OFF) randomly presented.

We used a block design, and each twenty-second task block was followed by a twenty-second resting block (Custead et al., 2017; Oh et al., 2017) (see Figure 2). The twenty-second block of either 5 cm/s, or 25 cm/s, or 65 cm/s, or “All ON”, or “All OFF” was randomized. The different velocities represented the different speeds of the air pressure pulses traveling (saltation) through channels (see Figure 1). For instance, the 5 cm/s represented that the air pressure pulses went through all channels sequentially within approximately 5 s in total (starting at channel 1, about 1 s at channel 2, 2 s at channel 3, 3 s at channel 4, and about 5 s at channel 5). The 25 cm/s meant that the pressure pulses went through all channels sequentially within approximately 2 s in total. The 65 cm/s represented that the air pressure pulses went through all channels sequentially within about 1.5 s in total. The “All ON” indicated that the air pressure pulses went through all channels simultaneously. The “All OFF” meant that no air pressure pulse went through all channels, which is equivalent to the resting period. During the fMRI scan, visual countdown task was used to maintain the participants’ vigilance using E-Prime 2.0 software (Psychology Software Tools, Pittsburgh, PA, United States). The participants were directed to pay attention to the number shown on the screen for only 0.5 s to minimize brain activation in the primary visual cortex. A declining countdown from 20 to 1 represented the remaining time in seconds for the tactile stimulation.

### Data Acquisition

All images were acquired on a 3.0 T Siemens Skyra whole-body MRI system (Siemens Medical Solutions, Erlangen, Germany) with a 32-channel head coil. A high-resolution T1-weighted three-dimensional anatomical scan was acquired using magnetization-prepared rapid gradient-echo sequences (MPRAGE) with the following parameters: TR/TE/TA = 2.4 s/3.37 ms/5:35 min, flip angle = 7°, field of view = 256 × 256 mm, spatial resolution = 1 × 1 × 1 mm^3^, number of slices = 192. Following the MPRAGE anatomical scan, three sessions of functional MRI (fMRI) scans were recorded using a T2^∗^-weighted echo planar imaging (EPI) sequence with the following parameters: TR/TE/TA = 2.5 s/30 ms/800 s, voxel size = 2.5 × 2.5 × 2.5 mm^3^, flip angle = 83°, number of slices = 41, number of volumes = 320.

The first TR pulse from the MRI scanner during fMRI data acquisition went through a Berkeley Nucleonics pulse generator (Model 645), which was in charge of sending triggers to the Galileo system to produce a velocity sequence every 40 s. The Galileo system generated a velocity condition for 20 s, then wait for 20 s to initiate the next velocity sequence. To reduce the effect of fatigue, we did three runs separately and gave optional breaks between runs. Each run consisted of 20 blocks, and 20 blocks consisted of four sets of randomly presented five conditions (5, 25, 65 cm/s, “All ON”, “All OFF”) (see Figure 2). Each run includes four blocks of 5 cm/s (80 s and 52 TR), four blocks of 25 cm/s (80 s and 52 TR), four blocks of 65 cm/s (80 s and 52 TR), four blocks of “All ON” (80 s and 52 TR), four blocks of “All OFF” (80 s and 52 TR). In total, each condition block lasted 960 s with 480 s condition segment and 480 s rest segment. Nineteen participants completed all three runs, while one participant only completed two runs.

### Data Analysis

The CONN toolbox (Whitfield-Gabrieli and Nieto-Castanon, 2012)^1^ was used for pre-processing all images and compute brain connectivity using both seed-based and region-of-interests (ROIs)-based approaches. The CONN toolbox used Statistical Parametric Mapping (SPM12^2^) toolbox to pre-process all image volumes, including functional realignment, structural segmentation and normalization, ART-based scrubbing, and smoothing. First, the functional data were realigned to the first scan and to correct for interscan head movement. The functional realignment process in the CONN toolbox automatically generated the first-level covariate with six rigid-body parameters that quantified the estimated motion for each participant and each run. The functional realignment covariate can be used in the GLM to regress out the residual movement-related effects from the time series. Second, the structural image was segmented into gray matter (GM), white matter (WM), and cerebrospinal fluid (CSF) in the individual participant’s space, and normalized to the Montreal Neurological Institute (MNI) space. The transformation matrix between the segmented MRI to MNI space was saved and used to coregister functional images to the normalized structural MRI. The Artifact Rejection Toolbox (ART^3^)-based scrubbing built-in CONN toolbox was applied to outlier detection and scrubbing to compute confound regressors using default parameters (global threshold: 9 stand-deviations above or below the mean, motion threshold: 2 mm translation and 2° rotation in any direction). The ART-based scrubbing technique detected an outlier if the largest voxel movement between volumes exceeded the thresholds. Only three outliners were identified and treated as nuisance regressors in the first-level GLM analysis. Finally, all coregistered fMRI data were smoothed with an isotropic Gaussian kernel of 8 mm full-width-at-half-maximum (FWHM).

The task-related functional connectivity was computed in the CONN toolbox. For each participant, CONN implemented CompCor to identify the top five principal components associated with segmented WM and CSF (Behzadi et al., 2007). These components were entered as confounds along with realignment parameters and nuisance regressors from ART-based scrubbing in the first-level GLM analysis. The residual time courses were linearly detrended (no despiking) and temporally filtered using a band-pass filter (0.008-0.09 Hz) during the denoising process in the CONN toolbox.

For the ROI-to-ROI analyses, we studied FC between ROIs for different velocities (5, 25, 65 cm/s, “All ON”). We computed the strength and significance of bivariate Pearson correlation among pairs of ten ROIs for each participant. Five bilateral ROIs (see Table 1) were created using MNI coordinates in the CONN toolbox and the MNI coordinates were based on our previous work (Custead et al., 2017; Oh et al., 2017). The ROI-to-ROI correlation coefficients were obtained by calculating all possible correlation coefficients between the time series of each pair of ROIs. For ROI-to-ROI connectivity, significant connections were identified by calculating the False Discovery Rate (FDR)-corrected two-sided p-value (*q*) at *q* < 0.05 thresholds for seed-level correction. The FDR seed-level correction applied FDR separately for each ROI and corrected across the multiple comparisons from having multiple target ROIs.

**TABLE 1:** Regions Of Interest.

**Name**	**Description**	**Coordinates in MNI space**		
		***X* (mm)**	***Y* (mm)**	***Z* (mm)**
**Left hemisphere**				
Left SI	ROI & Seed	−55	−19	24
Left SII	ROI & Seed	−48	−24	16
Left PPC	ROI	−56	−31	32
Left DLPFC	ROI	−27	32	36
Left Thalamus	ROI	−9	−17	6
**Right hemisphere**				
Right SI	ROI & Seed	56	−13	29
Right SII	ROI & Seed	48	−24	16
Right PPC	ROI	56	−31	32
Right DLPFC	ROI	30	35	34
Right Thalamus	ROI	10	−19	6

*MNI, Montreal Neurological Institute; ROI, region of interest; SI, primary somatosensory cortex; SII, supplementary somatosensory cortex; PPC, Posterior Parietal Cortex; DLPFC, dorsolateral Prefrontal Cortex.*

For seed-based FC analyses, a whole-brain approach was used to identify cortical areas that were differentially connected with bilateral SI and SII among four conditions (5, 25, 65 cm/s, and “All ON”). The four seeds (see Table 1) were chosen based on the literature (Lin and Kajola, 2003; Pastor et al., 2004; Reed et al., 2004; Dresel et al., 2008; Ackerley et al., 2012; Popescu et al., 2013; Custead et al., 2017; Oh et al., 2017; Yeon et al., 2017) because bilateral SI and SII were most consistently identified across imaging studies with different parameters. We chose to compute all possible cross-correlation coefficients between the time series of the seeds and all residual voxels in the brain, and then convert them to *Z*-scores. At the second-level analysis in the CONN toolbox, we compared FC patterns among different tactile stimuli. To control for multiple comparison, the CONN toolbox implemented the Cluster Size Statistic (CSS) (Ing and Schwarzbauer, 2014). FC maps between all voxel pairs for all participants under all conditions were generated, and then *T*-statistics were calculated between connectivity values taken under different velocity conditions. A cluster-forming threshold was set at voxel-level *p* < 0.001 and CSS only counted those with cluster-level FDR-corrected *p* < 0.05 as significant, which is a multiple-comparison correction at the whole-brain level to control the false discoveries among significant clusters below 5% rate (Friston et al., 1994).

## Results

### ROI-Based FC

**FIGURE 3:**
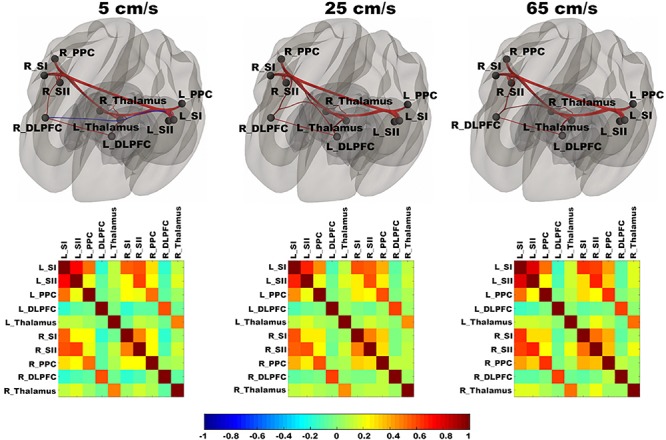
Shows ROI-to-ROI based connectivity maps (first row) and connectivity adjacent matrices (second row) for three velocities (5, 25, 65 cm/s). Total six region of interests (ROIs) include bilateral primary somatosensory cortex (L_SI and R_SI), bilateral supplementary somatosensory cortex (L_SII and R_SII), bilateral Posterior Parietal Cortex (L_PPC and R_PPC), bilateral dorsolateral Prefrontal Cortex (L_DLPFC and R_DLPFC), and bilateral thalamus (L_Thalamus and R_Thalamus). The color bar ranges from –1 to 1 and indicates the connectivity strength measured by Pearson’s correlation coefficients. On the connectivity maps, the ROIs are in block dots and positive connections are in red and negative connections are in blue. The thickness of the line is determined by the connectivity strength.

**FIGURE 4:**
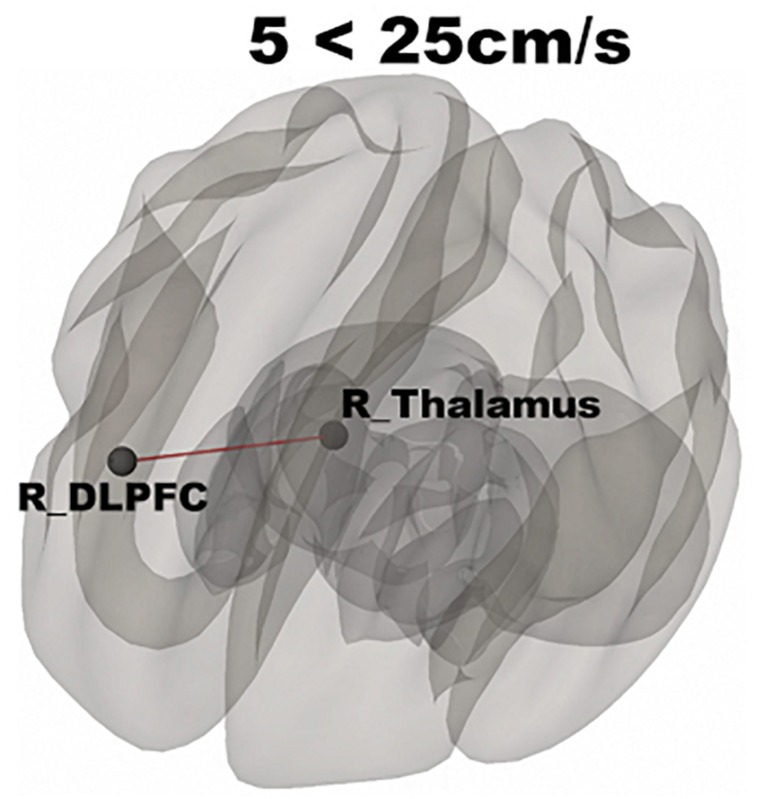
Shows significantly increased connectivity between the right thalamus and right DLPFC for 5 < 25 cm/s.

**FIGURE 5:**
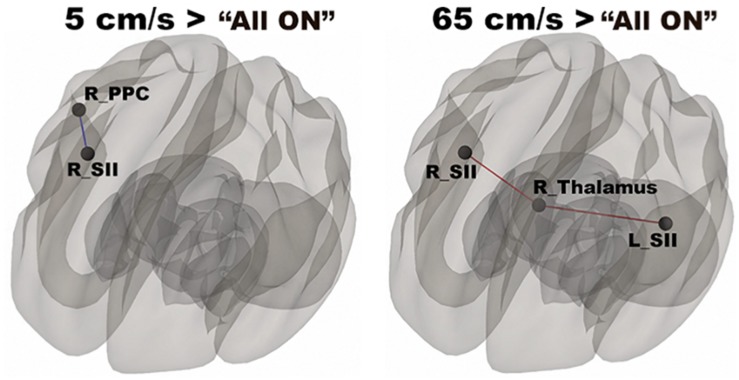
Shows significantly increased connectivity between the right SII and the right PPC for 5 cm/s > “All ON” and significantly increased connectivity between the right thalamus and the bilateral SII for 65 cm/s > “All ON”.

In Figure 3, functional networks for each velocity (5, 25, and 65 cm/s) were overlaid onto three-dimensional rendered brain on the first row and task-related FC matrices for each velocity were plotted on the second row. Comparing 5 and 25 cm/s task conditions, increased FC was identified between the right DLPFC and the right thalamus (see Figure 4). There is no significant difference of FC between 5 and 65 cm/s and between 25 and 65 cm/s for all ROI-to-ROI pairs. The contrast of 5 cm/s vs “All ON” task condition showed significant decreased FC between the right SII and the right PPC and the contrast 65 cm/s vs “All ON” task condition revealed increased FC between the right thalamus and the left SII and between the right thalamus and the right SII (see Figure 5).

### Seed-Based FC

**FIGURE 6:**
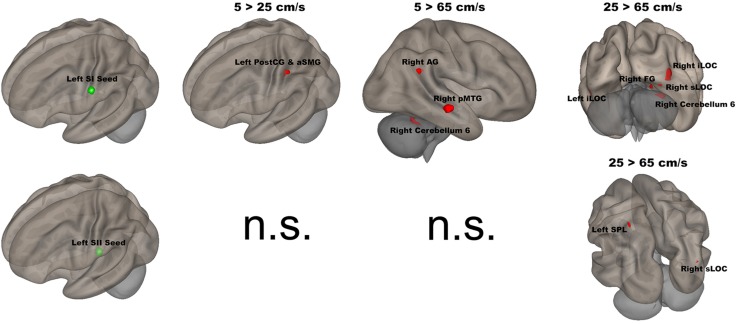
Shows the left SI (green sphere) and the left SII seeds (green sphere) overlaid on a standardized three-dimensional template and the significant seed-to-voxel results were presented on the right.

**FIGURE 7:**
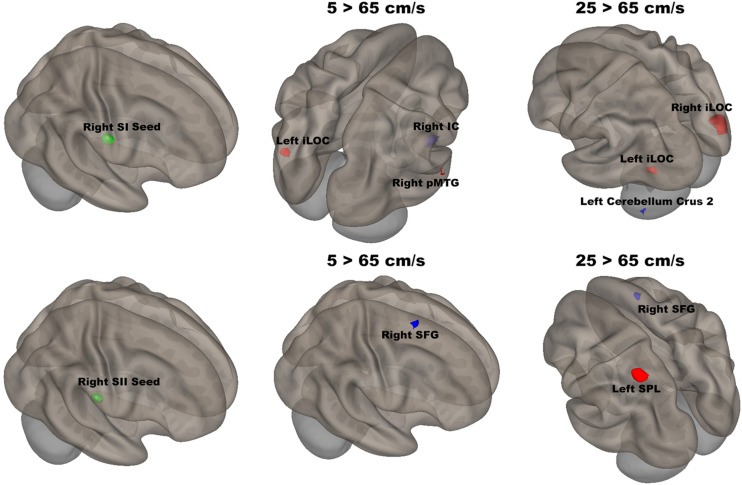
Shows the right SI (green sphere) and the right SII seeds (green sphere) overlaid on a standardized three-dimensional template and the significant seed-to-voxel results were presented on the right.

The seed-to-voxel analyses assessed FCs between four seed regions covering bilateral SI and SII and all other voxels in the brain (cluster size > 35, cluster size FDR-corrected). In Table 2 and Figure 6, for the left SI seed, results revealed increased FC in the left PostCG and left SMG for 5 > 25 cm/s, in the right pMTG, right cerebellum 6, and right AG for 5 > 65 cm/s, in the bilateral iLOC, right sLOC, right FG, right Cerebellum 6 for 25 > 65 cm/s. For the left SII seed, increased FCs were only in the left SPL and right sLOC for 25 > 65 cm/s. In Table 2 and Figure 7, for the right SI seed, increased FCs were shown in the left iLOC and right pMTG along with decreased FCs in the right IC for 5 > 65 cm/s, and increased FCs were also observed in the bilateral iLOC along with decreased FC in the left cerebellum crus II for 25 > 65 cm/s. For the right SII seed, decreased FC was present in the right SFG for both 5 > 65 cm/s and 25 > 65 cm/s. Additionally, increased FCs were shown in the left SPL and sLOC for 25 > 65 cm/s.

**TABLE 2:** Seed-to-voxel results of changes in functional connectivity related to each velocity.

**Region**	**Coordinates in MNI space**			**Voxels**
	**X (mm)**	**Y (mm)**	**Z (mm)**	
**1. Left SI seed**				
*Contrast: 5* > *25 cm/s*				
Left PostCG (35), aSMG (22)	−38	−36	42	57
*Contrast: 5* > *65 cm/s*				
Right pMTG	70	−22	−12	155
Right Cerebellum 6	34	−54	−24	73
Right AG	60	−52	26	64
*Contrast: 25* > *65 cm/s*				
Left iLOC (140)	−36	−78	2	140
Right iLOC (490), sLOC (138), FG (64)	40	−82	−6	692
Right FG (53), Right Cerebellum 6 (46)	34	−60	−22	99
**2. Left SII seed**				
*Contrast: 25* > *65 cm/s*				
Left SPL	−32	−52	68	42
Right sLOC	36	−84	18	42
**3. Right SI seed**				
*Contrast: 5* > *65 cm/s*				
Left iLOC	−42	−72	2	78
Right pMTG	64	−24	−4	40
*Contrast: 5* < *65 cm/s*				
Right IC	40	−4	−6	109
*Contrast: 25* > *65 cm/s*				
Left iLOC	−38	−78	0	96
Right iLOC	46	−82	−4	298
*Contrast: 25* < *65 cm/s*				
Left Cerebellum Crus II	−38	−66	−54	34
**4. Right SII seed**				
*Contrast: 5* < *65 cm/s*				
Right SFG	18	8	66	76
*Contrast: 25* > *65 cm/s*				
Left SPL	−30	−58	62	147
*Contrast: 25* < *65 cm/s*				
Right SFG	18	12	48	50

*PostCG, Postcentral Gyrus; aSMG, anterior Supramarginal Gyrus; pMTG, posterior Middle Temporal Gyrus; AG, Angular Gyrus; iLOC, inferior Lateral Occipital Cortex; sLOC, superior Lateral Occipital Cortex; FG, Fusiform Gyrus; SPL, Superior Parietal Lobule; IC, Insular Cortex; SFG, Superior Frontal Gyrus.*

## Discussion

The present study examined FC evoked by the orofacial tactile perception of velocity using fMRI in 20 neurotypical adults. This study attempted to identify FC evoked by novel saltatory pneumotactile stimuli using TAC-Cells with the Galileo system, which has not been reported previously. Our findings suggested that there were more similarities in ROI-to-ROI neuronal networks in the contralateral cortical areas (on the opposite side to the stimuli) and more differences in FC patterns in the ipsilateral cortical areas (on the same side as the stimuli). The 5 cm/s velocity evoked weaker FC between the right thalamus and the right DLPFC than did the 25 cm/s velocity, indicating that a velocity of 25 cm/s evoked stronger FC in the ipsilateral cortical regions. During the “All ON” condition, all TAC-Cells were stimulated simultaneously at 1 Hz, without varying velocity. The decreased FC between the right SII and right PPC for contrasting 5 cm/s with the “All ON” condition, and the increased FC between the right thalamus and bilateral SII for contrasting 65 cm/s with the “All ON” condition, demonstrated the FC pattern evoked by orofacial tactile perception of velocity. Our Seed-to-Voxel approach identified different cortical network patterns for each seed at various velocity contrasts (5 vs 25 cm/s, 5 vs 65 cm/s, and 25 vs 65 cm/s), suggesting that specialized FC patterns are responsible for discriminating different velocities of orofacial tactile stimuli.

### Effects of Velocity

In this study, the right side of the lower face was passively stimulated with air pulses from a spatial array of TAC-Cells, which produced a 2–3 mm deflection of the skin surface. Unlike the glabrous hand, the facial skin is non-glabrous and lacks Pacinian afferents (Barlow, 1987). Hairy skin also lacks Meissner afferents and contains both slowly conducting unmyelinated C-tactile afferents and fast-conducting myelinated Aβ fibers (Nordin, 1990; Ackerley et al., 2014). The Merkel afferents, a population of slow-adapting type 1 (SA1) afferents, were stimulated in the right lower face, and then their first-order afferents produced action potentials, which were carried by the first-order Aβ axons into the ipsilateral main sensory trigeminal nucleus to release neurotransmitters to the second-order afferents. The second-order afferents generated action potentials that were conducted by their axons into the contralateral pons, to the ventral trigeminal lemniscus. The action potentials arrived at the contralateral thalamus and released neurotransmitters to the third-degree afferents in the core of the ventral posteromedial nucleus (VPM) of the thalamus. Finally, the third-degree VPM afferents released neurotransmitters to the cortical neurons in the SI and initiated the higher-order processing of the spatiotemporal information about the tactile stimuli delivered to the face (Norrsell and Olausson, 1994; Lundblad et al., 2011).

Our ROI-to-ROI results presented greater similarity of FC across the three velocities in the contralateral hemisphere. The only difference in connection strength was between the right DLPFC, and the right thalamus, and the 5 cm/s velocity evoked weaker FC than did the 25 cm/s. The right DLPFC has been associated with many high-level functions, such as alerting, cognitive control, emotional regulation, and working memory (Critchley et al., 2001; Mannarelli et al., 2015; Schaal et al., 2017; Wang et al., 2018; Yang et al., 2018). A case report has shown that a patient with a thalamic lesion in the region of the right intralaminar nuclei was conspicuously slow, inflexible, and lacked concentration, suggesting that the right thalamus is critical for healthy cognitive functions (Van Der Werf et al., 1999). Our paradigm used a fixed block length. Thus, there were more air pulses delivered for the 25 cm/s blocks than for the 5 cm/s blocks. The 5 cm/s velocity, which had the lowest temporal density of tactile stimulation, required little attention during the task, leading to weaker FC between the attention region (right DLPFC) and important hub region (right thalamus). Interestingly, there were no differences in the FC connection strength between the right DLPFC and the right thalamus for contrasts of 5 vs 65 cm/s, or 25 vs 65 cm/s. This result indicated that a 65 cm/s velocity, the highest velocity used in this study, exceeded the optimal range of velocity. The optimal velocity range for moving tactile stimuli was 3–30 cm/s for the hand and 3–25 cm/s for the face (Langford et al., 1973; Whitsel et al., 1978, 1986; Dreyer et al., 1979; Essick et al., 1987, 1988; Edin et al., 1995). Additionally, the results of our previous study indicated that a velocity of 5 cm/s evoked the most extensive brain activation (Custead et al., 2017), indicating that there were sufficient data from three runs for the lowest temporal density of pneumotactile stimulation (5 cm/s) to evoke cortical activation. For 25- and 65-cm/s velocities, the higher temporal density of air-pulse stimulation did not elicit more BOLD responses in the brain, suggesting that adaptation or repetition-suppressing processes may play a role (Hollins et al., 1991; Popescu et al., 2013; Yang et al., 2014; Custead et al., 2017). Additionally, the 65-cm/s velocity exceeded the optimal range of velocity for the face and was processed differently in higher-order cortical levels of cortex in an animal study (Darian-Smith et al., 1984). High velocities have high temporal density, but have low perception accuracy (Lamb, 1983; Custead et al., 2017), whereas the 5-cm/s velocity might be processed as discrete stimuli to the facial skin, rather than as a constant motion across the skin (Wacker et al., 2011; Depeault et al., 2013). A recent study evaluated tactile pleasantness by stroking a soft brush over the skin and concluded that middle velocities, from 1 to 10 cm/s, were the preferred velocities, based on the pleasantness ratings (Ackerley et al., 2014). No participant in this study reported discomfort or pain sensation. Thus, pain-related neuronal networks did not influence our results.

In the “All ON” condition, the multichannel TAC-Cells were stimulated simultaneously. The contrasts of each velocity with the “All ON” condition revealed differences in FC strengths caused by the effects of the velocities of the tactile stimuli. For 5 cm/s vs “All ON” conditions, there was reduced FC between the right SII and the right PPC. Our previous results showed bilateral activation patterns when comparing 5 cm/s vs “All ON” (Custead et al., 2017). Our previous GLM results were limited to the strength of BOLD signals and could not determine the FC between a pair of cortical areas, while the present FC analysis allowed us to understand FC by calculation of Pearson’s correlation coefficients using time courses from pairs of cortical areas. A previous fMRI study has reported the representations of six body parts (face, fingers, legs, shoulders, lips, and toes) in the superior PPC (Huang et al., 2012). The right SII is connected reciprocally with the right (ipsilateral) SI (Karhu and Tesche, 1999). The “All ON” condition contains the highest temporal density of pneumotactile stimuli, and the fast Aβ fibers were used to carry the sensory information. The 5-cm/s velocity contained the lowest temporal density, and the slow C-tactile fibers were probably used to pass the sensory information. Therefore, the faster conduction from the peripheral nervous system (PNS) for the “All ON” condition allowed faster information flow between the right PPC and the right SII and led to stronger FC than the 5-cm/s velocity. For the 65 cm/s vs “All ON” conditions, there was increased FC between the right thalamus and bilateral SII. The 65-cm/s velocity is not optimal for evoking functional networks in brain supporting velocity encoding, but the 65-cm/s velocity elicited increased FC in the right thalamus. Our results support the role of the thalamus as an integrative hub for functional brain networks (Hwang et al., 2017). An early animal study found that SII receives substantial inputs from topographically appropriate regions within the ipsilateral ventrobasal nucleus and from the ipsilateral posterior group (Carvell and Simons, 1987), which indicated that SII in mice may complement the function of SI by helping to define the overall sensory context in which detailed tactile discriminations are made. Our findings suggested that the right SII was involved in both low and high velocities and might play an important role in discriminating the velocity of orofacial tactile stimuli (Carvell and Simons, 1987; Tommerdahl et al., 2005a). Moreover, there was no statistically significant difference in connection strength for the 25 cm/s vs “All ON” conditions. This unexpected finding indicated that the three velocities were processed differently at the CNS-level, and that different processes at the PNS level might be the driving force. Determining how the three velocities were processed in the PNS was beyond the scope of the present study.

Our Seed-to-Voxel analyses were limited to four seeds only, since the bilateral SI and SII were most commonly activated during tactile stimulation (Karhu and Tesche, 1999; Simoes and Hari, 1999; Backes et al., 2000; Lin and Kajola, 2003; Simoes et al., 2003; Inui et al., 2004; Blatow et al., 2007; Dresel et al., 2008; Eickhoff et al., 2008; Garraghty et al., 2009; Tommerdahl et al., 2010; Hu et al., 2012; Popescu et al., 2013; Vahdat et al., 2014; Venkatesan et al., 2014; Avanzini et al., 2016). Although more seeds could be added, the power of this study would have been affected due to the relatively small number of participants. The changes in FC patterns for different velocities and four seeds (bilateral SI and SII) suggested that each velocity is unique, and might be used based on the sensitivity and spatial specificity needed for the specific neurotherapeutic applications.

The left (contralateral) SI seed had stronger FC with the left PostCG/aSMG in the comparison of 5 vs 25 cm/s. The left PostCG/aSMG were reported to demonstrate significant increases in BOLD signals for 5 cm/s vs “All OFF” condition in our previous fMRI study (Custead et al., 2017). More specificially, the low velocity (5 cm/s) evoked both stronger FC and BOLD signals in the left PostCG/aSMG than did the mid-range velocity (25 cm/s). Lamb et al. reported that increases in stimulus velocity could lead to sufficient loss of spatiotemporal information to decrease discrimination accuracy (Lamb, 1983). FC was increased between the left SI seed and the right AG, right pMTG, and right cerebellum 6 for 5-cm/s velocity vs 65-cm/s velocity. The right AG has been related to numerical representation (Gobel et al., 2001). Our visual paradigm was a visual number countdown task. During the 5 cm/s blocks, the number countdown task-evoked time courses in the right AG that correlated with the time courses of BOLD signals in the left SI. The stronger FC between the left SI and the right FG for 5-cm/s velocity vs 65-cm/s velocity indicated that temporal accuracy was higher for the slowest velocity. The right pMTG has been shown to be involved in the frontoparietal network, which positively modulated cognitive tasks (Jolles et al., 2013). The low velocity evoked the largest spatial extent of activation in the comparison of 5 cm/s with the “All OFF” condition (Custead et al., 2017), which corresponded with the stronger FC between the left SI and right pMTG for 5-cm/s velocity vs 65-cm/s velocity. FC was increased between the left SI seed with the right FG, right sLOC, bilateral iLOC, and right cerebellum 6 for 25-cm/s velocity as compared to 65-cm/s velocity. The right FG, right sLOC, and bilateral iLOC cover the spatial extent of the occipital lobe, suggesting more involvement of visual attention for 25-cm/s velocity than for 65-cm/s velocity. The right cerebellum 6 was activated for both 5 vs 65 cm/s and 25 vs 65 cm/s. The right cerebellum 6 region is located at the right lobule VI of the cerebellum and is involved in the sensorimotor network (Bellebaum and Daum, 2007; Habas et al., 2009; Stoodley et al., 2012; Picerni et al., 2013; Guo et al., 2015), in line with our findings.

The left SII showed significantly increased FC only between the left SPL and right sLOC for 25 cm/s as compared to 65 cm/s. There were clear differences in FC patterns between the left SI and SII, in agreement with other studies (Backes et al., 2000). There were more similarities in FC patterns between 5 and 25 cm/s conditions in the left SII than for the left SI. Both velocities were within the optimal velocity range. The differences in FC between 25 and 65 cm/s conditions is likely to be driven by the 25-cm/s velocity, since there was no difference in FC for 5 vs 65 cm/s or 5 vs 25 cm/s. The left SII has been suggested to participate in the high-order processing of somatosensory stimuli (Backes et al., 2000), which was supported by our results.

There was significantly increased FC between the right SI seed and the left iLOC and right pMTG, as well as decreased FC between the right SI seed and right IC for 5 cm/s as compared to 65 cm/s. The right IC has been shown to be involved in inhibiting sensorimotor responses as part of the attention network (Corbetta and Shulman, 2002; Custead et al., 2017). The fastest velocity with the highest temporal density led to more repetition and required more control over response suppression. FC between the right SI seed and the bilateral iLOC was significantly increased, and FC between the right SI and the left cerebellum crus II was significantly decreased, for 25 cm/s as compared to 65 cm/s. The cerebellar involvement is consistent with the putative role of the cerebellum in feedforward control of sensory-guided movements at 5 cm/s (Custead et al., 2017).

The right SII showed significantly weaker FC with the right SFG for both 5 vs 65 cm/s and 25 vs 65 cm/s, but there was no difference for 5 vs 25 cm/s conditions. Thus, the noted FC difference was driven by the highest velocity. The right SFG plays a role in executive function, supporting bottom-up attention (Jolles et al., 2013). The increases in velocity required more bottom-up attention or alertness. Moreover, the right SII showed significantly stronger FC with the left SPL and weaker FC with the right SFG for 25 cm/s as compared to 65 cm/s. Somatosensory stimuli are processed in the left SPL, which is also involved in sensorimotor integration (Ruben et al., 2001). Weaker FC with the left SPL for 25 than for 65 cm/s might be due to the higher temporal density of the highest velocity stimulation. In other words, there are more somatosensory stimuli delivered in 20 s for the 65 cm/s than for either 5 or 25 cm/s stimuli.

### Laterality

Both contralateral and ipsilateral FC of both SI and SII during unilateral or bilateral activation have been reported in animal (Tommerdahl et al., 2005a, b) and human studies (Tommerdahl et al., 2006; Akselrod et al., 2017). Neurons in SII most often have bilateral receptive fields, unlike neurons in SI (Whitsel et al., 1969). Our ROI-to-ROI results showed stronger FC between the right thalamus and bilateral SII for 65 cm/s as compared to the “All ON” condition, supporting the view of the involvement of bilateral SII in unilateral stimulation. The present study demonstrated that FC was reduced between the right PPC and the right SII for 5 cm/s as compared to the “All ON” condition, suggesting that right SII activity evoked by the slow velocity is critical for neuronal encoding of orofacial tactile perception of velocity. We also observed changes in FC in both hemispheres, in alignment with our previous report on bilateral cortical responses (Custead et al., 2017). The different velocities evoked different brain connectivity patterns that were mostly noted in the right (contralateral) hemisphere, supporting the involvement of interhemispheric connections for complex pneumotactile stimulation.

### Limitations

This study had several limitations. First, a major limitation was that the imaging modality measured relatively slow hemodynamic responses, in the order of seconds. FMRI data can provide some indirect measures to decode how the sensory system perceives stimuli with different velocities. However, humans can make sensory decisions in less than 200 ms, which relies primarily on rapid synaptic neurotransmission on a time scale of milliseconds (Kohn et al., 2002). Thus, electrophysiology-based imaging approaches (i.e., MEG, EEG) are more suitable for studying the dynamic information of this rapidly changing system (Puts et al., 2019). Second, the relatively small sample size and wider age range of our participants could have limited the power of this study. Third, the FC analyses in the present study could not allow conclusions about the causal relationships between cortical regions and about whether the cortical network supporting higher-order processing of the facial tactile stimuli involved serial or parallel processing. Lastly, no behavioral measures were collected to assess individual differences in perception ability.

## Conclusion and Future Directions

In this study, cortical connectivity patterns associated with various tactile stimulation velocities were studied using fMRI, which has not been reported previously. Our results demonstrated both similarities and differences in the neuronal networks across the three velocities. Animal and human studies have shown that passively evoked sensory stimulation can enhance neuronal activity after stroke (Whitaker et al., 2007). Therefore, the present study has implications for applying passive pneumotactile stimuli, with various velocities, to bolster functional recovery during sensorimotor rehabilitation. For instance, if this is combined with physical therapy for stroke patients or brain-injury survivors, it might induce more brain plasticity during sensorimotor rehabilitation (Small et al., 2002; Luft et al., 2005; da Guarda and Conforto, 2014). In future, a large cohort study should investigate age- and sex-effects on the perception of velocity (Venkatesan et al., 2015). Moreover, the effect of placement of TAC-Cells (right side vs left side, etc.) on the face should be investigated. Finally, stroke survivors could be included as a comparison group in future studies. Rehabilitation protocols for stroke survivors can be designed using the Galileo system, and the efficacy thereof could be assessed using fMRI, or MEG, or both.

## Data Availability Statement

The raw data supporting the conclusions of this article will be made available by the authors, without undue reservation, to any qualified researcher.

## Ethics Statement

The study was reviewed and approved by the Institutional Review Board at University of Nebraska-Lincoln. Written informed consent was obtained from each participant in accordance with the Declaration of Helsinki.

## Author Contributions

YW proposed and performed connectivity analysis, and drafted the manuscript. SB contributed to the conception, design, and data collection of the study, and revising the manuscript critically for important intellectual content. RC and HO carried out the experiment and data collection. FS organized and pre-processed the data. All authors read and approved the submitted version and agree to be accountable for all aspects of the work.

## Conflict of Interest

The authors declare that the research was conducted in the absence of any commercial or financial relationships that could be construed as a potential conflict of interest.

## Footnotes

1 http://www.nitrc.org/projects/conn

2 https://www.fil.ion.ucl.ac.uk/spm/software/spm12/

3 http://www.nitrc.org/projects/artifact_detect/
